# LSTM-Based Estimation of Solar Energy Production Using Meteorological and Environmental Data: Karabük Case Study

**DOI:** 10.3390/s26103063

**Published:** 2026-05-12

**Authors:** Fatih Gultekin, Muhammet Tahir Guneser, Mehmet Zahid Yildirim

**Affiliations:** 1 Institute of Graduate Programs, Karabuk University, Karabuk 78050, Türkiye; 2 Department of Electronics and Communication Engineering, Istanbul Technical University, Istanbul 34475, Türkiye; guneserm@itu.edu.tr; 3 Faculty of Computer and Information Sciences, Karabuk University, Karabuk 78050, Türkiye; m.zahidyildirim@karabuk.edu.tr

**Keywords:** solar energy, meteorological data, environmental factors, LSTM

## Abstract

This study proposes a Long Short-Term Memory (LSTM)-based deep learning model for short-, medium-, and long-term forecasting of solar energy production. Approximately four years of hourly data from four photovoltaic power plants in Karabük were used. In addition to production data, meteorological and environmental variables were included through a multivariate forecasting approach. The model was tested under three scenarios at different time scales. Performance was evaluated using Root Mean Square Error (RMSE), Mean Absolute Error (MAE), Mean Absolute Percentage Error (MAPE) and coefficient of determination (R^2^) metrics. Results showed high prediction accuracy, particularly with seasonal data, where R^2^ values exceeded 0.90 in most cases. In forecasts based on monthly data, performance was more variable, and the shorter data window limited the model’s learning capacity. Long-term analyses indicated that the model successfully captured overall production trends and achieved high accuracy across all Photovoltaic (PV) systems. The findings also revealed that incorporating meteorological and environmental variables significantly improved prediction performance. In particular, air pollution parameters were effective in long-term production forecasting. Overall, the study demonstrates that Long Short-Term Memory (LSTM)-based models are reliable and effective tools for solar energy forecasting, with strong potential for energy planning and smart grid applications.

## 1. Introduction

The sustained increase in global energy demand, depletion of fossil fuel reserves, and international pressure to combat climate change have placed renewable energy sources at the center of energy policies [1,2]. Renewable energy is considered a fundamental component of today’s energy transition due to its direct derivation from natural resources, its low environmental impact, and its contribution to a sustainable energy supply [3,4]. Among these resources, solar energy stands out due to its low carbon footprint, modular structure, high scalability, and increasing economic viability in parallel with technological advancements [5,6]. The increasing popularity of photovoltaic (PV) systems in recent years, both on an individual and corporate scale, has further strengthened the role of solar energy in sustainable energy production [7].

Solar energy production is inherently variable and sensitive to external conditions. The performance of PV systems is determined not only by panel technology and installation specifications, but also by meteorological and environmental conditions [8,9]. Meteorological variables such as temperature, humidity, wind speed, precipitation, and cloud cover directly affect the energy conversion processes of solar panels [8]. When the effects of global warming on photovoltaic power generation are examined, it is clear that high temperatures are a key environmental factor that reduces cell efficiency [10,11]. In contrast, wind speed can enhance electricity generation by providing a cooling effect on the panel surface, and it can also help prevent performance losses by reducing dirt accumulation (soiling) on the surface [12,13].

Conversely, a high humidity level can reduce production performance by making it easier for dust and dirt particles to adhere to the panel surface. In this regard, ref. [14] explains the basic principles of humidity-related adhesion mechanisms, while studies such as [15], which examines climatic conditions in Oman, and [16], which analyzes the relationship between dust accumulation and humidity in Kenya, provide concrete evidence of these negative effects in different geographical contexts. Therefore, meteorological variables are among the key factors influencing not only the instantaneous performance of PV systems, but also their daily, seasonal, and annual performance trends [17,18].

In addition to meteorological parameters, environmental quality indicators also play a critical role in solar energy production [19]. Air quality indicators, particularly PM2.5 and PM10, accumulate on the panel surface, reducing optical transmittance and increasing maintenance requirements. The effects of dust accumulation on solar energy systems and the methods to mitigate these negative impacts have a critical place in the literature [20]. Studies conducted on a global scale indicate that aerosols and panel soiling lead to a reduction in solar energy efficiency worldwide [21]. The significant decreases in energy production capacity caused by particulate matter pollution have been identified as a fundamental environmental barrier [22]. Especially in regions experiencing high levels of air pollution, it has been determined that pollutants directly restrict the utilization of photovoltaic resources [23]. Finally, a holistic evaluation of environmental factors affecting the efficiency of photovoltaic systems is essential for optimizing system performance [24]. These effects can lead to a decrease in energy production and problems with system performance continuity. Therefore, reliable modeling and forecasting of solar energy production necessitates the combined evaluation of meteorological and environmental variables.

Accurate forecasting of solar energy production is essential not only for evaluating technical performance but also for supporting smart energy management and operational planning [25,26,27], facilitating renewable energy integration into smart grids [28], and improving capacity utilization from a techno-economic perspective [29,30]. Maintaining a balance between energy supply and demand, timely planning of maintenance processes, efficient facility operation, and supporting decision-making processes increase the need for reliable production forecasting models [31,32]. In addition to forecasting, recent studies have also shown that machine learning techniques can support fault detection and operational reliability in on-grid photovoltaic systems, further emphasizing the importance of data-driven approaches for PV system monitoring and management [33].

Recent research has increasingly emphasized the importance of accurate photovoltaic production forecasting for grid operation, system planning, and state estimation in modern power systems. In this context, PV generation forecasting is not only a technical prediction task but also a critical component of energy system monitoring, operational reliability, and smart grid decision-making. As PV penetration increases, production variability may directly affect grid stability, capacity planning, and real-time energy management. Therefore, robust data-driven forecasting models are needed to support reliable operation under changing environmental, meteorological, and system-level conditions [34,35].

In this context, data analytics and artificial intelligence-based approaches have emerged as powerful tools in monitoring, modeling, and predicting renewable energy systems in recent years. Energy production data generally exhibit nonlinear, non-stationary, and time-dependent behavior due to the combined effects of meteorological, environmental, and operational variables. Such characteristics often limit the ability of traditional statistical models to represent long-term dependencies, abrupt variations, and complex temporal interactions with sufficient accuracy. In this context, deep learning-based approaches have gained increasing importance in renewable energy forecasting because they can extract nonlinear and hierarchical representations directly from time-series data. Recent empirical studies have reported the strong predictive capability of deep learning architectures in solar power and photovoltaic generation forecasting [36,37]. Similarly, hybrid modelling frameworks incorporating deep learning have achieved promising results in renewable energy time-series forecasting [38], while deep learning techniques have also been employed effectively for the prognosis of solar thermal systems [39]. Furthermore, review evidence highlights the growing role of deep learning models in solar irradiance and photovoltaic power forecasting applications [40].

Among deep learning models, Long Short-Term Memory Networks (LSTMs) are particularly noteworthy for their ability to learn complex short- and long-term dependencies in time series. LSTM is a deep learning architecture designed to address gradient vanishing and information transfer problems encountered by traditional Recurrent Neural Networks (RNNs) when modeling long-term relationships. Its ability to selectively retain information from past steps via memory cells and gate mechanisms enables it to represent temporal patterns in a more robust and flexible way. In this respect, LSTM is an extremely suitable method for modeling the time-dependent effects of meteorological and environmental variables on solar energy production. Furthermore, studies in the literature on solar radiation and PV power estimation have reported that LSTM often achieves the lowest RMSE among individual models and exhibits better performance than classical machine learning methods [39,40,41]. In addition, many studies have shown that LSTM achieves a higher coefficient of determination (R^2^) and lower prediction errors than methods such as random forest regression (RFR), support vector machine (SVM), classical artificial neural network (ANN), and autoregressive integrated moving average (ARIMA) [36,37,38].

While numerous studies in the literature on solar energy production forecasting focus primarily on meteorological variables, a significant portion of these studies also examine other factors. Studies that integrate environmental quality indicators with actual production data within a single modeling framework are relatively rare. The scarcity of studies that consider air quality indicators such as PM2.5 and PM10 alongside meteorological variables like temperature, humidity, and cloud cover, and that support these findings with real PV production data from different production buildings, points to a significant research gap in this area. This gap is important not only for forecasting accuracy but also for developing scalable, application-oriented energy management models that are sensitive to site conditions.

As shown in Table 1, existing studies have demonstrated the effectiveness of deep learning, particularly LSTM-based models, in photovoltaic power forecasting. However, most of these studies are limited to either solar irradiance prediction, single-scale forecasting, or models based on a restricted set of meteorological variables. More importantly, the joint use of real PV production data with both meteorological and air quality variables remain largely unexplored. In particular, the integration of site-specific air pollution indicators (e.g., PM2.5, PM10, and gaseous pollutants) within a multivariate time-series forecasting framework is still limited in the literature. Therefore, there is a clear need for comprehensive, multi-source, and multi-scale forecasting models that can capture both atmospheric and environmental impacts on PV production.

Despite recent advances in deep learning-based renewable energy forecasting, several limitations remain in the existing literature. Many previous studies have mainly focused on solar irradiance, short-term PV power forecasting, or renewable energy time-series prediction with limited environmental inputs. However, the combined use of actual PV production data, meteorological variables, and comprehensive air quality indicators remains limited, especially in industrial–urban environments where gaseous and particulate pollutants may affect PV output. To address this gap, this study proposes a multivariate LSTM-based forecasting framework based on approximately four years of real hourly PV production data collected from four operational PV systems in Karabük, Türkiye. The model combines meteorological variables, including temperature, precipitation, sunshine duration, and cloudiness, with local air quality indicators such as PM10, PM2.5, CO, NO_2_, and SO_2_. Unlike many previous studies, the proposed model is evaluated under monthly, seasonal, and long-term forecasting scenarios, allowing PV production behavior to be examined across different time scales. The findings show that the model performs particularly well in seasonal and long-term forecasts, explaining more than 90% of the variance in most seasonal cases and approximately 85.88% to 93.80% of the variance in the long-term scenario. Therefore, the main scientific contribution of this study is the development and validation of a site-sensitive, multi-source, and multi-scale PV production forecasting framework that can support capacity planning, operational efficiency, grid stability, and sustainable energy management.

## 2. Methodology

**Figure 1:**
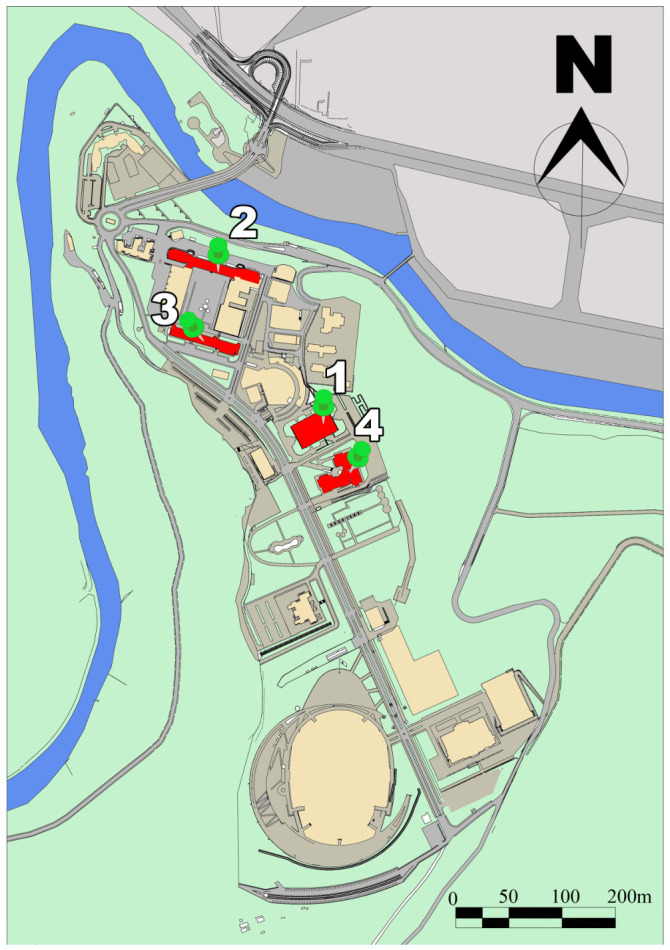
Solar panel placement locations.

This study is based on hourly production data obtained from photovoltaic (PV) systems in four different buildings on the Karabük University campus. The study area was chosen because it provides regular access to production data and allows for the evaluation of the effects of meteorological and environmental variables on PV production under real operating conditions. In this context, PV systems located in the buildings of the Faculty of Technology, the School of Foreign Languages, the Faculty of Economics and Administrative Sciences, and the Faculty of Theology were examined. The examined facilities used PV modules of the same brand and nominal power; however, the number of panels, installed power, and inverter configuration differed from building to building. The placement of the PV panels is shown in Figure 1.

When the four facilities are considered together, it has been determined that the system consists of 3741 PV panels, with a total DC installed capacity of 1028.775 kWp and a total AC installed capacity of 1010 kWe. This structure corresponds to a distributed PV generation infrastructure of approximately 1 MW on a campus scale. The research design is based on evaluating model performance on seasonal, monthly, and long-term time scales. The technical specifications of the systems are presented in Table 2.

**Figure 2:**
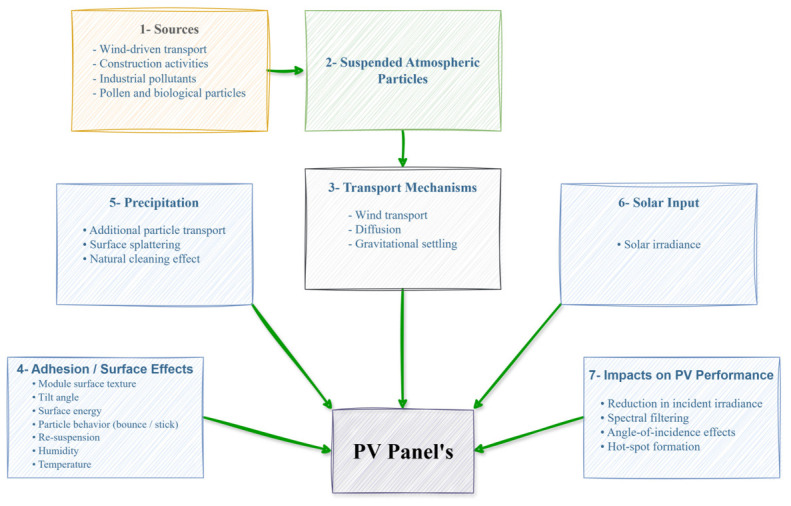
Factors affecting solar panel production.

Meteorological and environmental variables were considered together because they represent atmospheric conditions that can directly or indirectly affect PV generation. Figure 2 presents the conceptual framework showing the direct and indirect effects of these variables on PV generation performance [12]. This framework served as the basis for the variable set and the multivariate modeling approach used in the study.

The dataset consists of hourly PV production data from four locations, along with meteorological and environmental variables expected to influence this production. The PV production data comprises hourly production records obtained from the SCADA automation systems of the respective buildings and was procured through the Karabük University Renewable Energy Engineering Research and Application Center. Meteorological data were obtained from relevant meteorological stations through the Meteorology Directorate, while environmental data were obtained from three different air quality monitoring stations in Karabük through official consultations with the Directorate of Environment, Urbanization, and Climate Change.

The initial feature pool consisted of 27 candidate variables representing meteorological and environmental conditions. Subsequently, a hybrid feature selection strategy was adopted. First, Pearson correlation analysis was used as a preliminary step to examine linear relationships and identify redundant variables. However, since PV generation is governed by nonlinear and time-dependent dynamics, correlation alone was deemed insufficient for final selection. Therefore, a multi-criteria evaluation was conducted, where each feature was tested individually within the LSTM framework and model performance was assessed using RMSE, MAE, MAPE, and R^2^ metrics. Furthermore, domain information regarding the physical effects of meteorological and air quality parameters on PV performance was also included. Based on this multi-criteria evaluation, 10 variables that consistently contributed to the prediction performance were selected for the final model. A detailed list of variables and measurement points is given in Table 3.

Prior to modeling, PV production data and meteorological and environmental variables were aligned on a common one-hour time axis using timestamps. Missing values were imputed using linear interpolation, and problematic records that could compromise the analysis’s integrity were removed from the dataset. Subsequently, the final set of independent variables determined through a hybrid feature selection strategy was used in the modeling phase. Data normalization was applied to prevent variables with different scales from negatively affecting model performance during training. In this context, Z-score standardization was used for both input variables and the target variable.

(1) $$z=\frac{x-\mu }{\sigma }$$

where μ and σ represent the mean and standard deviation of the variable, respectively.

In the next stage, the data structure was transformed into time-series input–output sequences that enabled the prediction of future production values from past observations. The natural flow of the time series was preserved when creating the training and test structures, and random splitting was not applied. Long Short-Term Memory (LSTM) was used to estimate solar energy production with the determined model and variables. LSTM was preferred because of its ability to learn short- and long-term dependencies in hourly PV production [44].

The Long Short-Term Memory (LSTM) network is designed to capture temporal dependencies through a memory cell and gating mechanisms. At each time step t, the LSTM unit is governed by the following equations:

(2) $$i_{t}=\sigma (W_{i}.\left[h_{t-1},x_{t}\right]+b_{i})$$

(3) $$f_{t}=\sigma (W_{f}.\left[h_{t-1},x_{t}\right]+b_{f})$$

(4) $$o_{t}=\sigma (W_{o}.\left[h_{t-1},x_{t}\right]+b_{o})$$

(5) $${\bar{C}}_{t}=tanh(W_{c}.\left[h_{t-1},x_{t}\right]+b_{c})$$

(6) $$C_{t}=f_{t}⊙C_{t-1}+i_{t}⊙{\bar{C}}_{t}$$

(7) $$h_{t}=o_{t}⊙tanh(C_{t})$$

where $x_{t}$ represents the input vector at time step t, $h_{t}$ is the hidden state, and $c_{t}$ denotes the cell state. The operators σ and tanh represent the sigmoid and hyperbolic tangent activation functions, respectively, while ⊙ denotes element-wise multiplication. W and b are the corresponding weight and bias parameters. In this study, the input vector $x_{t}$ consists of an 11-dimensional feature set, including 10 selected meteorological and environmental variables and one lagged production value. Thus, the model follows a multivariate autoregressive structure where the future production value is estimated as

(8) $${\hat{y}}_{t+1}=f(x_{t},h_{t})$$

where f(·) represents the nonlinear mapping learned by the LSTM network. All this modeling and experimental analysis was performed using the Deep Learning Toolbox in MATLAB R2019b.

No explicit detrending or differencing operation was applied before model training. This choice was made to preserve the original temporal structure of the PV production series, including seasonal patterns, long-term tendencies, and natural production variability. Since the objective of the study was to evaluate the ability of LSTM to learn temporal dependencies from real multivariate PV production data, the model was trained on the standardized but non-differenced time series. Z-score standardization was applied to reduce scale-related effects, while the chronological order of the observations was preserved throughout the training and testing process.

**Figure 3:**
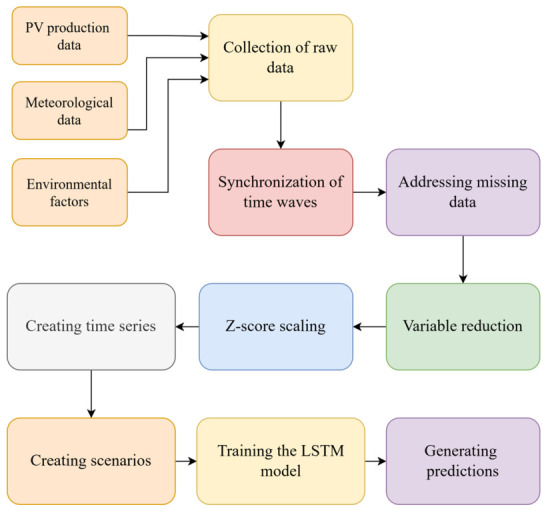
Conceptual framework of the proposed PV production forecasting system.

Figure 3 illustrates the overall conceptual framework of the proposed solar energy forecasting system, showing the data flow from input sources to prediction output. The model operates within a multivariate structure that integrates photovoltaic production data with meteorological variables (e.g., temperature, cloudiness, precipitation) and environmental indicators such as air quality parameters. By combining these heterogeneous data sources, the framework captures both physical and environmental factors affecting solar energy generation. The figure also highlights the sequential nature of the forecasting process, where past observations are utilized to estimate future production values within a data-driven modeling pipeline.

**Figure 4:**
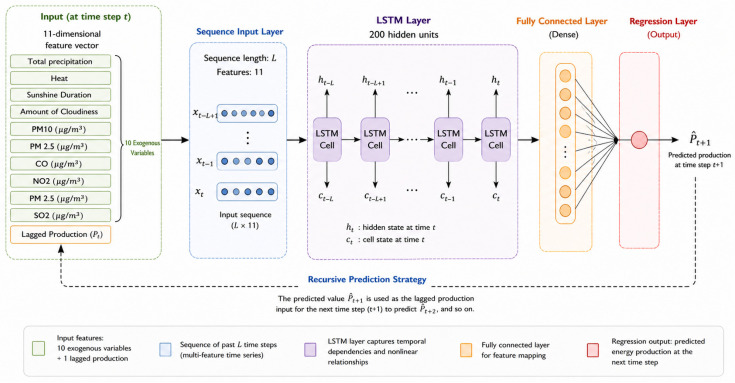
Architecture of the proposed LSTM-based forecasting model.

The proposed architecture consists of a sequence input layer that accepts 11-dimensional time-series inputs, an LSTM layer with 200 hidden neurons, a fully connected layer, and a regression layer. The model output is the energy production value at the next step. This structure makes the proposed method an autoregressive multivariate LSTM prediction model that uses historical production information and selected exogenous variables together. The model was trained using the Adam optimization algorithm [45]. During training, the maximum number of epochs was set to 100, the initial learning rate to 0.001, and the gradient threshold to 0.1. The learning rate was controlled via piecewise scheduling, and the Mean Squared Error (MSE) loss function was minimized during training. Predictions were generated using a recursive strategy that progresses stepwise during the testing phase. The model’s architectural structure is shown in Figure 4.

The performance of the developed model was evaluated using RMSE, MAE, MAPE, and R^2^ criteria. Performance analysis was conducted separately for each building. Results were reported at short, medium, and long-term evaluation levels. Calculations for the evaluation criteria are shared in Equations (9)–(12).

(9) $$RMSE=\sqrt{\frac{1}{n}\sum_{i=1}^{n}(y_{i}-{\hat{y}}_{i}{)}^{2}}$$

(10) $$MAE=\frac{1}{n}\sum_{i=1}^{n}|y_{i}-{\hat{y}}_{i}|$$

(11) $$MAPE=\frac{100}{n}\sum_{i=1}^{n}|\frac{y_{i}-{\hat{y}}_{i}}{y_{i}}|$$

(12) $$R^{2}=1-\frac{\sum_{i=1}^{n}(y_{i}-{\hat{y}}_{i}{)}^{2}}{\sum_{i=1}^{n}(y_{i}-\bar{y}{)}^{2}}$$

RMSE represents the root mean square error; MAE represents the mean absolute deviation between the predicted and actual values; MAPE represents the relative error as a percentage; and R^2^ indicates the extent to which the model explains the observed variation. Using these measures together, model performance was evaluated in terms of both error level and explanatory power.

Although alternative sequence models such as GRU, Temporal Convolutional Networks, and transformer-based architectures have recently shown promising performance in time-series forecasting, LSTM was selected in this study for several reasons. First, the primary aim of the study was not to benchmark different deep learning architectures, but to evaluate the forecasting capability of a recurrent multivariate model using real PV production data together with meteorological and environmental variables. Second, LSTM is well suited for learning both short- and long-term temporal dependencies in hourly PV generation data through its memory-cell and gating mechanisms. Third, compared with transformer-based models, LSTM generally requires fewer data and computational resources, which makes it appropriate for medium-sized real-world datasets such as the one used in this study. Nevertheless, the comparison of LSTM with GRU, TCN, and attention-based transformer architectures is recognized as an important direction for future work.

During the preparation of this article, ChatGPT (OpenAI; model: GPT-5.4 Thinking) was used to improve the language, clarity, and readability of the text. Its use was limited to editorial support only. All literature reviews, source selection, interpretation of findings, citation verification, and final editing were performed by the authors.

## 3. Results

In this section, the performance of the proposed LSTM-based model was evaluated through experimental studies conducted at different time scales. Analyses were performed under three different scenarios: short-term (1 week of forecasting with 1 month of data), medium-term (1 week of forecasting with 3 months/seasonal data), and long-term (1 year of forecasting with approximately 4 years of data). Model performance was analyzed separately for each PV system using RMSE, MAE, MAPE, and R^2^ metrics. In addition to the overall performance evaluation, feature contribution and robustness analysis were conducted to quantify the relative impact of meteorological and environmental variables on forecast accuracy and to assess the consistency of these effects across different stations.

### 3.1. Feature Contribution and Robustness Analysis

This subsection presents a detailed analysis of feature contribution and robustness in order to better understand the role of individual variables and variable groups in the prediction performance of the proposed model. The feature selection process in this study was based on a hybrid strategy combining filter-based methods (correlation analysis), wrapper-based evaluation (model performance), and domain knowledge regarding the physical relationships between atmospheric conditions and PV production.

In the initial stage, Pearson correlation analysis was employed as a preliminary filtering step to provide an initial understanding of linear relationships between variables and to identify potentially redundant features. However, since PV production is governed by nonlinear and time-dependent dynamics, correlation analysis alone was not considered sufficient for final feature selection. Therefore, a wrapper-based evaluation using the LSTM model was conducted to assess the actual contribution of each feature in a predictive context.

In this context, two complementary analyses were conducted. First, each feature was evaluated individually to assess its standalone predictive capability across different stations. Second, group-based experiments were performed to investigate the relative contribution of meteorological and environmental variable sets. These analyses aim to reveal not only the importance of each variable but also the robustness and consistency of their effects across different production systems.

Table 4 presents the prediction performance obtained when each feature is used individually as input to the model. The results show that all features provide relatively similar performance levels, with R^2^ values generally ranging between 0.83 and 0.95 across different stations. This indicates that no single variable dominates the prediction process, and the model benefits from a combination of multiple features rather than relying on a single input.

Among the evaluated variables, meteorological features such as sunshine duration, temperature, and cloudiness consistently demonstrate strong predictive capability across all PV systems. In particular, sunshine duration yields one of the lowest error values and highest R^2^ scores, highlighting its direct physical relationship with solar energy production.

On the other hand, environmental variables such as PM10, PM2.5, CO, and NO_2_ also contribute to prediction performance, although their standalone effects appear slightly weaker compared to core meteorological variables. Despite this, their performance remains comparable, suggesting that these variables provide complementary information rather than acting as primary drivers.

Another important observation is the consistency of feature performance across different stations. The relative ranking of features and their corresponding performance metrics remain stable across all four locations, indicating that the selected features are robust and not site-specific. This supports the generalizability of the proposed feature set.

Table 5 presents the results of group-based experiments designed to evaluate the relative contribution of different variable groups. The findings clearly indicate that meteorological variables alone provide strong predictive performance, achieving high R^2^ values across all PV systems (up to 0.92). This confirms that meteorological conditions constitute the primary driving factors of solar energy production.

When environmental variables from individual stations are considered separately, their performance is slightly lower but still significant. In particular, variables from the 75th Anniversary and Safranbolu stations yield competitive results, suggesting that air quality parameters contain useful information related to PV production.

However, when all environmental variables are combined into a single input set, a noticeable decrease in performance is observed. This suggests that environmental variables alone are insufficient to fully explain production variability and may introduce noise when used without meteorological context.

The most important finding emerges when meteorological and environmental variables are combined. Although the performance improvement is not dramatic, the combined model consistently achieves more balanced and stable results across all PV systems. This indicates that environmental variables provide complementary information that enhances the model’s ability to capture complex and nonlinear relationships.

Furthermore, the consistency of results across different stations demonstrates the robustness of the feature groups. The relative contribution patterns remain similar regardless of station characteristics, confirming that the selected variable groups are not dependent on a specific location.

Overall, these findings highlight that meteorological variables play a dominant role, while environmental variables act as supportive factors that improve model robustness and generalization.

### 3.2. Seasonal (3-Month Data) Forecast Results

Table 6 present the results of a one-week forecast performed using three months (seasonal) of data for four different production stations. Overall, the findings indicate that the model captures seasonal variability with high accuracy.

When examining the results for the Faculty of Economics and Administrative Sciences presented in Table 6, it is observed that the R^2^ value increased to 0.9563, particularly during the winter of 2024, while performance relatively decreased (R^2^ = 0.6829) during some summer periods (e.g., summer 2022). This indicates that increased production fluctuations during the summer months make model predictions more difficult.

Examining the Faculty of Technology results presented in Table 7, it is noteworthy that the model exhibited a particularly high performance during the summer of 2023 (R^2^ = 0.9714). Similarly, R^2^ values were generally at or above 0.94 throughout 2024. These results demonstrate that the model can produce successful predictions even in systems with high production capacity.

Table 8 shows the results for the School of Foreign Languages. The table shows that the model achieved very high accuracy, particularly in 2024 (up to R^2^ = 0.9730). However, it was observed that MAPE values increased during certain periods, indicating higher relative error rates during specific time intervals.

**Figure 5:**
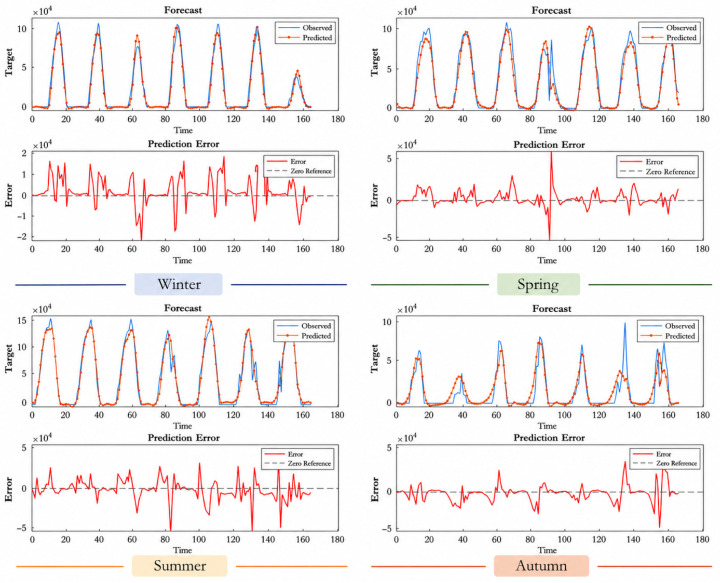
Economics faculty seasonal results forecast and error graphs.

**Figure 6:**
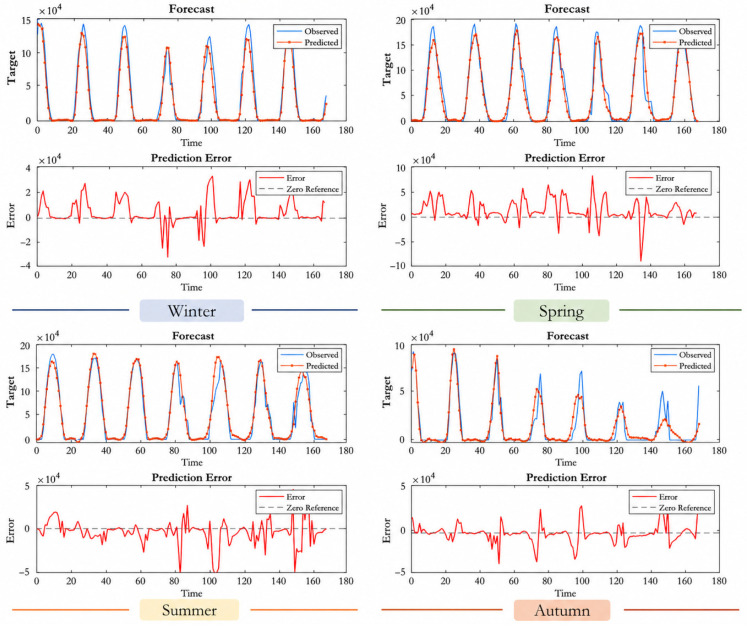
Prediction and error graphs for seasonal results of the Faculty of Technology.

**Figure 7:**
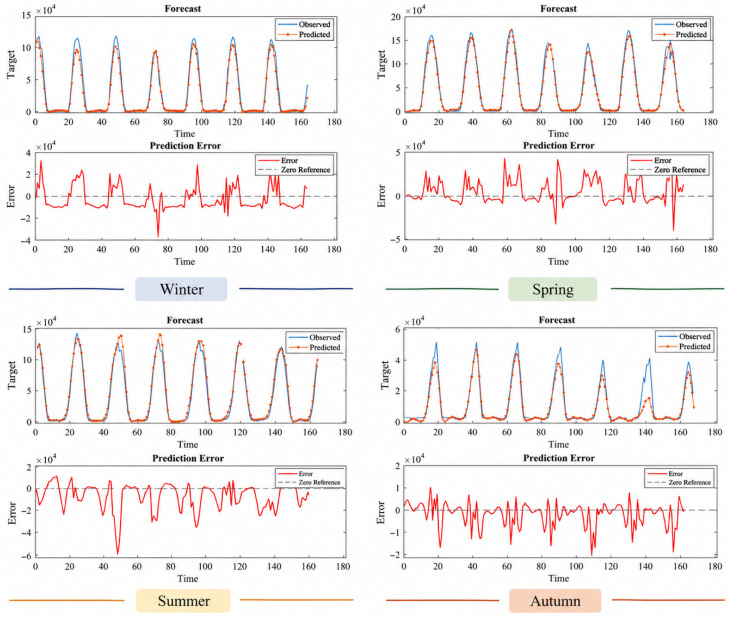
Prediction and error graphs for seasonal results of the School of Foreign Languages.

**Figure 8:**
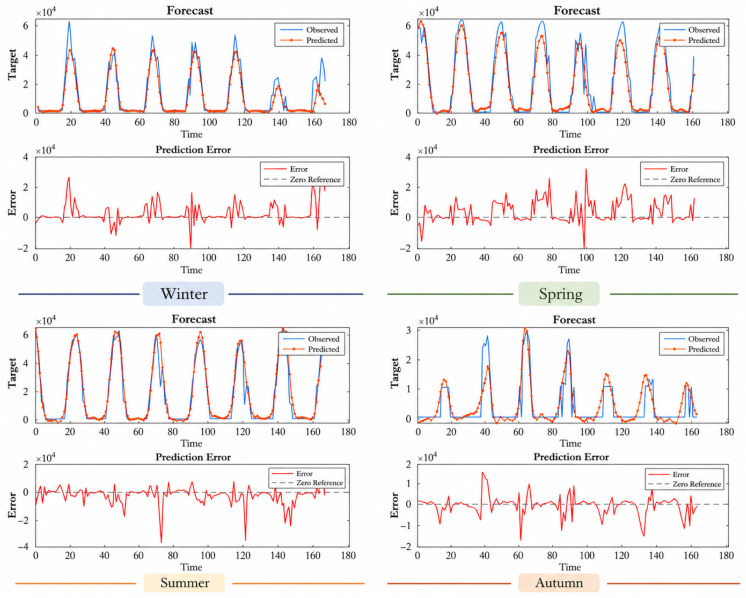
Prediction and error graphs for seasonal results of the Faculty of Theology.

The results for the Faculty of Theology, presented in Table 9, show that the model exhibits consistent performance despite having a lower installed power compared to other stations. The R^2^ value of 0.9436 obtained during the summer of 2023 is particularly noteworthy. However, a decrease in performance was observed during some autumn periods (e.g., autumn 2024, R^2^ = 0.6947). Examining the prediction and error graphs in Figure 5, Figure 6, Figure 7 and Figure 8, it can be seen that the model outputs generally track the actual production values. In the error graphs, it was observed that deviations increased during periods of sudden production changes, while the error remained at very low levels during stable production periods. This indicates that the LSTM model successfully learns temporal dependencies but is less sensitive to sudden changes.

Examining the seasonal forecast graphs presented in Figure 5, it can be seen that the LSTM model generally follows actual production values successfully. The agreement between the forecast curve and actual values is particularly high during the spring and winter periods. This indicates that the model exhibits strong generalization capabilities in periods with more stable production patterns. During the summer months, production values increase, leading to occasional deviations from the forecast curve. Examination of the error graphs reveals that the error variance is higher during this period compared to other seasons. In the autumn, model performance improves again, and error levels decrease. Overall, the model captures seasonal variations to a large extent at this station, but error rates increase during periods of high volatility.

The results shown in Figure 6 reveal that the model performed quite successfully at this station. The agreement between the predicted and actual value curves is particularly noteworthy during the spring and summer periods. The more pronounced production patterns during these periods positively influenced the model’s learning process. When examining the error graphs, a generally low and balanced error distribution is observed. However, sudden error peaks are seen in some short time intervals. This situation may be due to the model’s inability to fully reflect sudden meteorological changes or environmental effects. The model accurately captured the general trend during winter and autumn, but the relative error increased at low production levels. Nevertheless, the overall performance is quite high, and the model produces reliable predictions for this station.

Figure 7 shows that the model’s prediction performance is generally successful, but fluctuations are more pronounced during certain periods. The agreement between the prediction curve and the actual values is particularly high during the spring and summer months. However, significant deviations are observed in the error graphs, especially during periods of sudden production changes. This indicates that production data has a more variable structure and that the model struggles to fully capture this variability. During the winter period, error rates are observed to increase relatively due to lower production levels. In the autumn period, the model’s performance is observed to rebalance, and predictions converge more closely to actual values. Overall, the model is successful at this station, but it produces errors with higher variance compared to other stations.

Examining the results in Figure 8, the model shows quite consistent performance at this station. The agreement between the prediction curves and the actual production values is particularly high during the spring and summer periods. The error graphs generally show a low-level and balanced distribution. This may be due to the relatively low production variability in this system with lower installed capacity. However, sudden increases in errors are observed in some short periods. It is noteworthy that there was a slight decrease in model performance and an increase in deviations in the prediction curve during the autumn period. Nevertheless, the overall error levels are acceptable. It can be stated that the model produced stable and reliable predictions for this station.

### 3.3. Monthly (1-Month Data) Forecast Results

Table 10 presents a statistical summary (mean, minimum, and maximum values) of the prediction performance results for all measurement stations, based on 1 month of data. This table enables a more general assessment of the model’s short-term predictive performance. According to the results, the average R^2^ values ranged from 0.73 to 0.83 across all PV systems. The highest average success was achieved at the Foreign Languages PV system (R^2^ = 0.8291), while the lowest average performance was observed at the Theology station (R^2^ = 0.7387). This indicates that there are certain performance differences between stations in short-term predictions.

When the minimum R^2^ values are examined, it is noteworthy that there are periods when quite low performance is obtained, especially in the Technology (0.3004) and Theology (0.1521) stations. This indicates that the short data window makes it difficult for the model to learn sufficient patterns in some periods, leading to significant performance decreases. In contrast, the fact that the maximum R^2^ values exceed 0.94 at all stations (e.g., 0.9851 for Foreign Languages) indicates that the model can make predictions with quite high accuracy under appropriate conditions.

When evaluated using error metrics, it is observed that the average RMSE and MAE values differ across stations. The highest average error values were observed at the Technology PV system (RMSE ≈ 20,016.48, MAE ≈ 12,481.91), while the lowest error values were obtained at the Theology station (RMSE ≈ 8328.79, MAE ≈ 5301.36). This situation can be attributed to the differences in the production scales and data distributions of the stations.

When MAPE values are examined, the average error rate ranges from 42% to 57%. The fact that maximum MAPE values reach quite high levels in some stations (e.g., 184.48% for Technology and 131.25% for Economics) indicates that the relative error increases significantly, especially during periods of low production values. In contrast, the fact that minimum MAPE values drop to 15–20% indicates that quite successful predictions were achieved during certain periods. Overall, the model trained on monthly data is usable for short-term forecasting, but its performance shows high variance. In particular, the wide difference between the minimum and maximum values indicates that model performance can vary significantly over time. This suggests that forecasts made with a short data window are more sensitive to the dataset’s structure and seasonal patterns.

**Figure 9:**
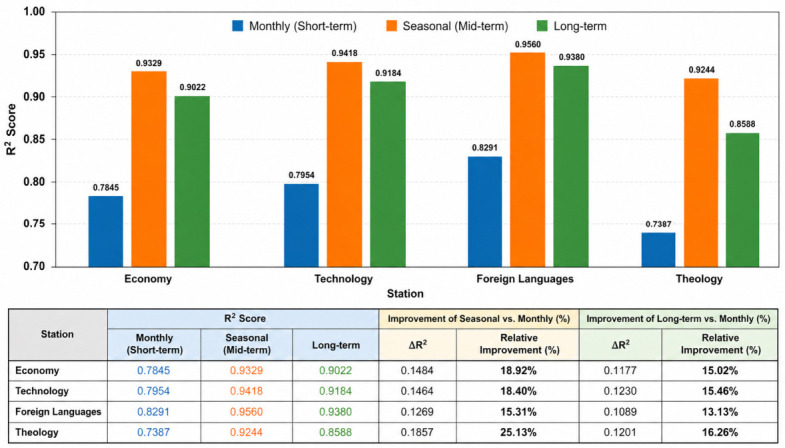
Comparative Performance of LSTM Model Across Stations and Forecasting Scenarios (R^2^).

To provide a clearer comparison of model performance across different forecasting scenarios, a visual representation of R^2^ values is presented in Figure 9. The figure illustrates the performance differences between monthly (short-term), seasonal (mid-term), and long-term forecasting across all PV systems. As shown, the model consistently achieves higher accuracy in seasonal and long-term scenarios compared to the monthly case. This indicates that increasing the temporal window allows the LSTM model to better capture underlying production patterns and temporal dependencies.

Among the stations, the Foreign Languages building exhibits the highest performance across all scenarios, while the Theology building shows relatively lower accuracy, particularly in the short-term scenario. The relative improvements between scenarios highlight that seasonal forecasting provides approximately 15–25% improvement over monthly forecasting, while long-term forecasting also shows a consistent increase in prediction accuracy. These findings confirm that model performance is strongly influenced by the length and structure of the training data, and that longer time windows enhance the model’s generalization capability.

### 3.4. Long-Term (All Data) Forecast Results

Table 11 presents the 1-year forecast results obtained using approximately 4 years of data. This scenario was designed to evaluate the model’s capacity to learn long-term patterns.

An examination of the results reveals that high R^2^ values were achieved at all stations. The School of Foreign Languages achieved the highest performance with R^2^ = 0.9380, while the Faculty of Technology (R^2^ = 0.9184) and the Faculty of Economics (R^2^ = 0.9022) also had very successful results. The Faculty of Theology, however, achieved a lower but acceptable performance compared to the other stations with R^2^ = 0.8588.

When RMSE and MAE values are examined, it is seen that the errors are proportional to the system capacity. While absolute error values increase in systems with higher installed capacity, the model shows consistent relative performance across R^2^ and MAPE metrics. These results demonstrate that the proposed LSTM model can successfully learn not only short-term fluctuations but also long-term production trends.

## 4. Discussion

In this study, the performance of the proposed LSTM-based model was comprehensively evaluated through experimental analyses conducted at different time scales. The findings reveal that the model successfully learns both short-term fluctuations and long-term production trends. However, it was determined that performance varies with the data window used, seasonal variability, and station-based production characteristics.

The model shows the most balanced and consistent performance with seasonal (3-month) data. R^2^ values are largely above 0.80 at all four stations and reach 0.95 in some periods. The literature reports that R^2^ values above 0.80 ensure high reliability in LSTM-based PV prediction models [42,43,46]. This shows that the model predicts accurately when trained on sufficiently long data with seasonal patterns. Pronounced production patterns in spring and summer improved learning and increased prediction accuracy. However, sudden production changes in some summer and autumn periods raised error values, revealing the model’s limited sensitivity to high-frequency fluctuations.

In short-term forecasts using monthly data, model performance was observed to be more variable compared to the seasonal scenario. In particular, when the amount of data is limited, the model fails to learn sufficient patterns and produces low R^2^ values in some months. However, it is noteworthy that the model achieves high accuracy values during periods when the production pattern is more stable (e.g., summer months). These results show that short data windows directly affect model performance, and that LSTM models generalize more effectively over longer time intervals. Furthermore, the significantly high MAPE values in some months are due to increased relative error, especially at low production levels, which partially limits the reliability of short-term forecasts.

Long-term analyses show that training the model on the entire dataset produces more stable and accurate predictions. R^2^ values above 0.85 for all stations indicate that the model successfully learns long-term production trends. The high R^2^ values obtained, particularly at the Foreign Languages School and Technology Faculty stations, demonstrate the model’s strong generalization capability, even across systems with varying capacities and production profiles. While RMSE and MAE varied proportionally with system capacity, relative performance metrics (R^2^ and MAPE) were more consistent across stations.

When evaluated on a station basis, it is observed that the model’s performance depends not only on the data length but also on the physical characteristics and production stability of the production system. In stations with high installed capacity, production fluctuations are more pronounced, leading to increased error values, while the model produces more stable results in smaller-scale systems. However, a common finding across all PV systems is an increase in errors during periods of sudden production changes. This indicates that despite the inclusion of meteorological and environmental variables in the model, some sudden effects could not be fully modeled.

The inclusion of meteorological and environmental variables in the model is a significant factor in improving forecast performance. Specifically, the model has successfully learned the direct impact of variables such as temperature, sunshine duration, and cloud cover on production. In addition, the inclusion of PM2.5, PM10, and other air quality indicators has contributed to improved performance, particularly in long-term forecasts. This confirms that environmental factors significantly impact solar energy production and cannot be ignored. These findings highlight the importance of accurately capturing both deterministic and potentially uncertain aspects of PV production.

Another important practical implication of the proposed forecasting model is its potential use in anomaly detection and fault diagnosis. Although photovoltaic production can be directly measured through monitoring systems, predictive models such as LSTM provide an expected production profile based on historical and environmental conditions.

By comparing the predicted values with the actual measurements, significant deviations may indicate abnormal system behavior. Such discrepancies can be associated with sensor malfunctions, data quality issues, or faults in system components such as inverters or panels. Therefore, the proposed model can be extended to serve as a reference model for detecting anomalies and identifying potential faults in photovoltaic systems.

While this study focuses on forecasting performance, integrating anomaly detection and fault diagnosis mechanisms based on prediction errors represents a promising direction for future research.

From a practical perspective, uncertainty-aware forecasting is particularly important for grid management and energy planning. While point forecasts provide a single expected value, they do not reflect the variability and potential deviations caused by sudden meteorological or environmental changes. In photovoltaic systems, such fluctuations may significantly affect operational decisions.

Although the proposed model in this study focuses on deterministic point forecasting, uncertainty-aware approaches such as prediction intervals, quantile regression, and probabilistic deep learning methods can provide additional insights for risk-sensitive decision-making. Incorporating these methods would require modifications in the model structure and training strategy and is therefore considered as an important direction for future research.

Overall, the proposed LSTM-based approach was found to be a flexible model capable of generating highly accurate predictions across different time scales. The model’s strongest performance was observed with seasonal data, while its weakest was in monthly analyses with short data windows. Long-term analyses, however, demonstrated the model’s ability to successfully capture general production trends. These findings highlight the significant potential of the proposed approach for energy production planning, maintenance process optimization, and decision support system development.

Although the proposed LSTM model achieved high performance, particularly in seasonal and long-term forecasting scenarios, its ability to handle abrupt non-stationary changes and regime shifts is limited. Sudden weather anomalies, unexpected production drops, maintenance interruptions, or policy-driven operational changes may generate patterns that are not sufficiently represented in the training data. This was reflected in the error graphs, where deviations increased during periods of sudden production variation. Therefore, future studies should consider anomaly-aware forecasting frameworks, regime-switching models, attention mechanisms, or transformer-based architectures to improve robustness against abrupt changes in PV generation dynamics.

## 5. Conclusions

In this study, an LSTM-based deep learning approach for predicting photovoltaic solar energy production at different time scales was developed and evaluated through comprehensive experimental analyses. Analyses conducted using approximately four years of hourly data showed that the model was successful in learning both short-term fluctuations and long-term production trends.

The findings show that model performance varies depending on the length and time scale of the data used. Specifically, the model produced the most stable and accurate results when forecasting with 3-month (seasonal) data. In quantitative terms, R^2^ values ranged between approximately 0.73 and 0.83 in the monthly (short-term) scenario, increased to approximately 0.92–0.96 in the seasonal scenario, and reached approximately 0.85–0.94 in the long-term forecasting scenario. These results indicate that extending the temporal window significantly improves model performance. In addition, seasonal forecasting provided an improvement of approximately 15% to 25% over monthly forecasting, while long-term forecasting also yielded consistent performance gains.

This indicates that the LSTM architecture’s ability to learn temporal dependencies becomes more effective with sufficiently long data containing seasonal patterns. Conversely, performance was more variable with forecasts made using 1-month data, and lower accuracy values were observed in some periods. This result reveals that short data windows limit the model’s generalization ability. In the long-term forecasting scenario, training the model on the entire dataset yielded more stable, accurate results. The high R^2^ values obtained at all stations demonstrate that the model successfully captures general production trends. This shows that the proposed approach is suitable not only for short-term forecasting but also for long-term energy production planning.

Station-based analyses have shown that model performance depends not only on data length but also on the characteristics of the production system. Among the analyzed stations, the Foreign Languages building exhibited the highest prediction accuracy across all scenarios, while the Theology building showed relatively lower performance, particularly in the short-term scenario. Systems with high installed capacity exhibit more pronounced production fluctuations, leading to increased error values, while systems with more stable production profiles yield more consistent results. Furthermore, it was determined that across all PV systems, errors increased during periods of sudden production changes.

One of the study’s key contributions is the adoption of a multivariate forecasting approach that incorporates both meteorological and environmental variables. The results show that parameters such as temperature, sunshine duration, and air pollution significantly improve model performance. This finding underscores the need to consider environmental factors in solar energy production forecasting.

In conclusion, the proposed LSTM-based model has been shown to produce reliable and highly accurate forecasts across different timescales and to be an effective method for modeling solar energy generation using meteorological and environmental data. However, the variability observed in short-term monthly forecast results indicates that limited data windows restrict the model’s ability to learn stable temporal patterns. Since the primary objective in this study was to evaluate model performance under real-world conditions using independent station-based datasets, data enhancement, transfer learning, or cross-field training strategies were not applied. This approach, while preserving the inherent characteristics of each photovoltaic system, also limits the model’s ability to generalize under conditions of data scarcity. Therefore, future studies should enhance the model’s sensitivity to abrupt changes by integrating attention mechanisms, comparing the proposed approach with alternative architectures such as GRU, TCN, and Transformer-based models, and investigating cross-field learning, transfer learning, and data enhancement techniques to improve robustness and prediction performance, particularly in short-term prediction scenarios.

## Figures and Tables

**Table 1:** Comparison of Previous Forecasting Studies and the Proposed Approach.

Ref.	Main Focus	Model/Approach	Forecasting Target	Input Variables	Forecast Scale	Main Difference from This Study
[36]	Solar power prediction	Comparative deep learning architectures	Solar power	Solar power-related variables	Forecasting-oriented	Compares deep learning architectures but does not focus on comprehensive meteorological and air quality integration.
[37]	Hourly PV power forecasting	Conditional GAN and Bi-LSTM	PV power	Time-series PV-related inputs	Hourly	Provides hourly PV forecasting but does not include comprehensive local air quality indicators.
[38]	Renewable energy time-series forecasting	DWT–SARIMA–LSTM	Wind power	Wind-related time-series variables	Short-term	Shows the effectiveness of hybrid LSTM-based forecasting, but it is not focused on PV production or air quality effects.
[40]	Solar irradiance and PV power forecasting	Review of deep learning models	Solar irradiance and PV power	Meteorological/irradiance-related variables	Review	Summarizes deep learning models but does not provide a site-specific real PV production forecasting application.
[42]	PV solar energy prediction	ASOA-optimized LSTM	PV energy	Temperature, humidity, cloud cover	Decomposition-based/multi-scale	Uses optimized LSTM for PV prediction but does not include comprehensive air quality indicators.
[43]	PV power forecasting	RNN–LSTM	PV power	PV/weather-related variables	Hour-ahead	Uses real PV plant data but is limited to hour-ahead forecasting and does not include air pollution variables.
This study	PV production forecasting using meteorological and environmental data	Multivariate LSTM	Actual PV energy production	Temperature, precipitation, sunshine duration, cloudiness, PM10, PM2.5, CO, NO_2_, SO_2_	Monthly, seasonal, and long-term	Combines real hourly PV production data from four operational PV systems with meteorological and comprehensive air quality indicators in a multi-scale forecasting framework.

**Table 2:** Technical specifications of PV panels.

Order	Building (Short Number)	Number of Panels	Panel Power	Total DC Installed Power	Total AC Installed Power
1	Faculty of Economics and Administrative Sciences **(1)**	1202	275 W	330.55 kWp	290 kWe
2	Faculty of Technology **(2)**	1136	275 W	312.4 kWp	310 kWe
3	School of Foreign Languages **(3)**	980	275 W	269.5 kWp	270 kWe
4	Faculty of Theology **(4)**	423	275 W	116.325 kWp	140 kWe
	**Total**	**3741**	**275 W**	**1028.775 kWp**	**1010 kWe**

**Table 3:** Variable Table.

Variable Group	Variable	Measuring Point
Meteorological	Total precipitation	Meteorological data
Meteorological	Temperature	Meteorological data
Meteorological	Sunshine duration	Meteorological data
Meteorological	Amount of cloudiness	Meteorological data
Environmental	PM10 (µg/m^3^)	Karabük 75th Anniversary Station
Environmental	PM2.5 (µg/m^3^)	Karabük 75th Anniversary Station
Environmental	CO (µg/m^3^)	Safranbolu Station
Environmental	NO_2_ (µg/m^3^)	Safranbolu Station
Environmental	PM2.5 (µg/m^3^)	Tören Alanı Station
Environmental	SO_2_ (µg/m^3^)	Tören Alanı Station

**Table 4:** Individual feature-based performance evaluation across different PV systems using the LSTM model.

Features	Economy				Technology				Foreign Languages				Theology			
	RMSE	MAE	MAPE	R^2^	RMSE	MAE	MAPE	R^2^	RMSE	MAE	MAPE	R^2^	RMSE	MAE	MAPE	R^2^
Total Precipitation	12,621.77	7554.50	36.47	0.89	14,514.38	8801.82	45.85	0.90	9418.59	6208.68	26.71	0.95	6782.33	3838.10	29.97	0.83
Temperature	12,611.01	7572.23	37.87	0.90	14,383.98	8504.29	48.17	0.90	9646.19	6326.95	27.53	0.95	6648.28	3683.34	30.17	0.84
Sunshine Duration	12,132.24	7084.13	34.80	0.90	13,019.22	7393.40	38.00	0.92	9992.53	6394.71	25.91	0.94	6114.90	3231.71	28.85	0.86
Cloudiness	12,471.87	7542.36	36.33	0.90	14,435.33	8811.46	48.60	0.90	9032.01	6045.48	25.61	0.95	6552.62	3661.49	29.58	0.85
PM10	12,452.05	7490.18	37.52	0.90	14,088.03	8471.01	46.74	0.91	9966.41	6556.58	28.61	0.95	6630.19	3680.34	30.50	0.84
PM2.5	12,413.66	7399.64	37.30	0.90	14,356.63	8711.88	45.98	0.90	9379.68	6076.17	26.02	0.95	6675.98	3677.83	31.01	0.84
CO	12,698.28	7685.58	38.34	0.89	15,110.67	9289.48	46.89	0.90	10,046.09	6550.35	27.25	0.94	6774.22	3767.89	32.03	0.84
NO_2_	12,564.12	7474.05	36.83	0.90	13,858.62	8329.99	44.58	0.91	9035.82	5930.42	24.86	0.95	6573.70	3666.88	30.11	0.84
PM2.5	13,377.55	8053.25	42.25	0.88	14,647.64	8808.44	48.09	0.90	9004.59	5957.98	24.77	0.95	6747.11	3759.30	31.12	0.84
SO_2_	12,508.61	7445.85	38.19	0.90	14,331.84	8626.98	46.26	0.91	9038.43	5860.16	26.79	0.95	6916.10	3897.32	32.69	0.83
Average	12,844.84	7679.62	38.19	0.89	14,622.03	8763.85	46.56	0.90	9724.99	6313.58	27.15	0.94	6738.47	3739.28	31.00	0.84

**Table 5:** Group-based feature contribution analysis for meteorological and environmental variable sets across different PV systems.

		Meteorological	75th Anniversary Station	Safranbolu Station	Tören Alanı Station	All Environmental Station	Meteo. + All Env. Station
Economy	RMSE	12,167	12,438	12,495	15,799	16,171	16,021
	MAE	6951	7346	7216	8723	9027	8921
	MAPE	34.89	38.14	38.00	39.77	42.30	40.93
	R^2^	0.90	0.90	0.90	0.83	0.82	0.83
Technology	RMSE	12,921	14,683	13,755	17,938	18,455	17,896
	MAE	7258	8722	7915	9648	10,083	9678
	MAPE	35.69	49.56	44.45	49.46	49.76	46.46
	R^2^	0.92	0.90	0.91	0.85	0.84	0.85
Foreign Languages	RMSE	10,155	9159	10,257	13,934	14,225	14,750
	MAE	6047	5974	6395	7607	8053	8368
	MAPE	26.32	26.76	27.82	32.80	33.35	34.06
	R^2^	0.94	0.95	0.94	0.88	0.88	0.87
Theology	RMSE	6209	6750	6645	7904	8191	8065
	MAE	3224	3710	3690	4252	4416	4444
	MAPE	31.06	32.17	30.30	33.98	36.58	37.83
	R^2^	0.86	0.84	0.84	0.77	0.76	0.78

**Table 6:** Economics faculty forecast results.

	RMSE	MAE	MAPE	R^2^
2022-Winter	12,134.36	6671.50	41.49	0.8546
2022-Spring	11,632.23	7182.86	26.01	0.9347
2022-Summer	21,958.59	13,199.84	56.41	0.6829
2022-Autumn	9329.07	5760.14	36.56	0.7907
2023-Winter	9034.17	4963.42	38.18	0.8355
2023-Spring	15,139.85	9926.19	36.10	0.8295
2023-Summer	12,078.71	8302.23	20.62	0.9479
2023-Autumn	7020.51	4349.83	41.87	0.8907
2024-Winter	7504.08	5010.80	26.51	0.9563
2024-Spring	11,232.51	6858.26	25.57	0.9329
2024-Summer	11,987.53	7594.37	21.74	0.9458
2024-Autumn	12,802.45	7542.89	53.84	0.7021

**Table 7:** Prediction results from the Faculty of Technology.

	RMSE	MAE	MAPE	R^2^
2022-Winter	13,276.07	6966.16	43.07	0.8303
2022-Spring	18,937.76	12,416.20	34.06	0.9212
2022-Summer	19,693.48	11,692.07	41.70	0.9079
2022-Autumn	11,106.01	6805.79	40.50	0.8353
2023-Winter	12,535.75	7783.75	33.22	0.9208
2023-Spring	20,613.81	12,723.07	42.22	0.9070
2023-Summer	11,187.62	7127.01	16.54	0.9714
2023-Autumn	9601.21	6074.07	55.14	0.9007
2024-Winter	11,339.35	6812.26	29.76	0.9611
2024-Spring	16,170.05	10,506.75	27.21	0.9407
2024-Summer	15,529.58	9602.17	23.22	0.9418
2024-Autumn	9816.40	5716.24	45.03	0.8436

**Table 8:** Prediction results from the School of Foreign Languages.

	RMSE	MAE	MAPE	R^2^
2022-Winter	12,187.00	6587.00	57.46	0.8576
2022-Spring	14,613.38	8767.41	21.61	0.9349
2022-Summer	17,267.10	11,074.11	45.87	0.9109
2022-Autumn	9661.70	5671.59	36.72	0.8340
2023-Winter	13,684.38	7864.35	40.48	0.8624
2023-Spring	21,669.73	12,802.35	40.29	0.8734
2023-Summer	14,219.32	8544.58	21.93	0.9301
2023-Autumn	7791.21	5179.01	47.69	0.9133
2024-Winter	10,311.71	6355.42	26.43	0.9730
2024-Spring	14,023.33	9793.76	22.04	0.9560
2024-Summer	15,815.03	11,048.75	49.45	0.9471
2024-Autumn	6913.30	4744.50	27.42	0.9186

**Table 9:** Prediction results from the Faculty of Theology.

	RMSE	MAE	MAPE	R^2^
2022-Winter	5603.97	3372.26	38.68	0.8375
2022-Spring	6835.05	4456.94	21.79	0.9311
2022-Summer	8090.76	5149.15	31.60	0.8809
2022-Autumn	5151.96	3031.15	31.72	0.7760
2023-Winter	6644.91	3901.56	40.22	0.7967
2023-Spring	9966.63	6082.45	34.30	0.8685
2023-Summer	5740.43	3805.69	16.44	0.9436
2023-Autumn	4716.56	3066.93	27.28	0.8373
2024-Winter	6862.88	3756.67	37.54	0.8602
2024-Spring	7677.78	5345.23	29.28	0.9244
2024-Summer	5900.91	3467.71	24.13	0.9472
2024-Autumn	4080.05	2583.28	29.02	0.6947

**Table 10:** Average results from all measurement stations over a monthly statistical period.

		Economy	Technology	Foreign Languages	Theology
RMSE	Avg.	16,821.91	20,016.48	16,070.07	8328.79
	Min.	7327.01	1862.97	2789.44	2429.87
	Max.	31,150.57	33,729.44	32,661.02	14,165.32
MAE	Avg.	10,699.45	12,481.91	10,451.88	5301.36
	Min.	4195.86	989.90	1907.33	1456.10
	Max.	22,390.52	22,586.72	22,442.29	10,191.09
MAPE	Avg.	48.41	56.65	44.26	42.59
	Min.	20.72	19.15	15.57	19.11
	Max.	131.25	184.48	80.89	68.80
R^2^	Avg.	0.7845	0.7954	0.8291	0.7387
	Min.	0.5482	0.3004	0.5369	0.1521
	Max.	0.9552	0.9666	0.9851	0.9447

**Table 11:** Long-term forecast results for all stations.

Short Number	RMSE	MAE	MAPE	R^2^
Building (1)	12,200	7070	34.1491	0.9022
Building (2)	13,300	7550	39.0329	0.9184
Building (3)	10,100	6190	25.8798	0.9380
Building (4)	6290	3310	31.2056	0.8588

## Data Availability

In this study, publicly available datasets were analyzed. Meteorological data were obtained from relevant meteorological stations through the Meteorology Directorate, while environmental data were obtained from three different air quality monitoring stations in Karabük through official consultations with the Directorate of Environment, Urbanization, and Climate Change. Experimental data obtained by the authors can be provided to the relevant author upon reasonable request.
